# Influence of Season, Population and Individual Characteristics on the Prevalence of *Leptospira* spp. in Bank Voles in North-West Germany

**DOI:** 10.3390/biology10090933

**Published:** 2021-09-18

**Authors:** Elisabeth Schmidt, Anna Obiegala, Christian Imholt, Stephan Drewes, Marion Saathoff, Jona Freise, Martin Runge, Jens Jacob, Anne Mayer-Scholl, Rainer G. Ulrich, Martin Pfeffer

**Affiliations:** 1 Institute of Animal Hygiene and Veterinary Public Health, University of Leipzig, 04103 Leipzig, Germany; elisabeth.schmidt@vmf.uni-leipzig.de (E.S.); anna.obiegala@vetmed.uni-leipzig.de (A.O.); 2 Institute for Plant Protection in Horticulture and Forests, Julius Kühn-Institute (JKI), 48161 Münster, Germany; christian.imholt@julius-kuehn.de (C.I.); jens.jacob@julius-kuehn.de (J.J.); 3 Institute of Novel and Emerging Infectious Diseases, Friedrich-Loeffler-Institut (FLI), Federal Research Institute for Animal Health, 17493 Greifswald-Insel Riems, Germany; stephan.drewes@fli.de (S.D.); rainer.ulrich@fli.de (R.G.U.); 4 Lower Saxony State Office for Consumer Protection and Food Safety, 26203 Wardenburg, Germany; marion.saathoff@laves.niedersachsen.de (M.S.); jona.freise@laves.niedersachsen.de (J.F.); 5 Lower Saxony State Office for Consumer Protection and Food Safety, 30173 Hannover, Germany; martin.runge@laves.niedersachsen.de; 6 German Federal Institute for Risk Assessment, 12277 Berlin, Germany; anne.mayer-scholl@bfr.bund.de

**Keywords:** leptospirosis, *Clethrionomys glareolus*, *L. interrogans*, *L. kirschneri*, *L. borgpetersenii*, MLST

## Abstract

**Simple Summary:**

Leptospirosis is a worldwide emerging zoonotic disease. Clinical symptoms in humans range from mild flu-like symptoms to severe clinical disease with kidney failure and multiple organ dysfunction. Infections occur after contact with infected animals or through water and soil contaminated by urine of infected animals. Cases are mostly linked to occupational risk groups such as field workers or farmers, but contact with pets and recreational activities like fresh water sports also lead to a higher exposure risk. This study was conducted to evaluate the prevalence and species distribution of *Leptospira* in bank voles in Germany. We analyzed the DNA of 1817 kidney samples and detected a mean prevalence of 7.5% with the following pathogenic *Leptospira* species: *L. interrogans*, *L. kirschneri*, and *L. borgpetersenii*. The individual infection risk in bank voles depended on season, body weight and abundance of bank voles. Even if leptospirosis case numbers in Germany are low, our study shows that pathogenic *Leptospira* spp. are present and a potential source for human infection, which should be recognized by clinicians and veterinarians.

**Abstract:**

Leptospirosis is a worldwide zoonotic disease with more than 1 million human cases annually. Infections are associated with direct contact to infected animals or indirect contact to contaminated water or soil. As not much is known about the prevalence and host specificity of *Leptospira* spp. in bank voles (*Clethrionomys glareolus*), our study aimed to evaluate *Leptospira* spp. prevalence and genomospecies distribution as well as the influence of season, host abundance and individual characteristics on the *Leptospira* prevalence. Bank voles, which are abundant and widely distributed in forest habitats, were collected in the years 2018 to 2020 in North-West Germany, covering parts of North Rhine-Westphalia and Lower Saxony. The DNA of 1817 kidney samples was analyzed by real-time PCR targeting the *lipl32* gene. Positive samples were further analyzed by targeting the *secY* gene to determine *Leptospira* genomospecies and multilocus sequence typing (MLST) to determine the sequence type (ST). The overall prevalence was 7.5% (95% confidence interval: 6.4–8.9). *Leptospira interrogans* (83.3%), *L. kirschneri* (11.5%) and *L. borgpetersenii* (5.2%) were detected in bank voles. Increasing body weight as a proxy for age increased the individual infection probability. Only in years with high bank vole abundance was this probability significantly higher in males than in females. Even if case numbers of human leptospirosis in Germany are low, our study shows that pathogenic *Leptospira* spp. are present and thus a persisting potential source for human infection.

## 1. Introduction

The genus *Leptospira* comprises a large group of spirochetal bacteria, which can be genetically divided into 66 different species or serologically into 24 serogroups with more than 300 serovars [1]. Additionally, *Leptospira* can be assigned to different sequence types (ST) on a genetic base [1,2]. Species of the genus *Leptospira* can also be divided according to their pathogenicity into pathogenic (P) and saprophytic (S) clades and two subclades each (P1, P2, S1, S2). For many species the pathogenicity is not clear yet and they are listed as likely pathogenic [3].

Leptospirosis is a potentially fatal zoonosis with more than 1 million human cases worldwide annually [4]. Highly endemic areas are located in tropical and subtropical regions and are characterized by poor hygiene, heavy rainfall and flooding [5]. Most cases occur in Latin America (35.8%), followed by South Asia (12.9%) [6]. Recent studies show that leptospirosis is also prevalent in highly developed countries with moderate climate [7,8,9,10,11,12,13]. The European Centre for Disease Prevention and Control (ECDC) recorded over 1000 confirmed cases in the European Union in the year 2019 [14]. Case numbers in Europe and particularly in Germany are increasing from an incidence of 0.04 per 100,000 inhabitants in 2003 to 0.14 in 2020, with peaks in the years 2014 and 2019 (each ≥ 0.19) [15].

Nearly every mammalian species can either be a main reservoir host or an accidental host for *Leptospira* spp. [16,17]. In main reservoir hosts (e.g., rodents, large and small ruminants, swine, horses, dogs and cats), *Leptospira* spp. persist in the proximal renal tubular epithelium [18]. Mostly without exhibiting clinical symptoms, main reservoir hosts shed *Leptospira* spp. via urine over a long time-period. The transmission is promoted by environmental conditions such as ground moisture and host population size [19,20,21].

Infections of accidental hosts with pathogenic *Leptospira* spp. occur after direct contact with infected animals or indirectly through contact with water or soil, which is contaminated by the urine of infected animals. Portals of entry are mucosal membranes of conjunctival, oral or genital surfaces as well as cuts and abrasions [22]. Leptospirosis outbreaks in humans are often connected to flooding and heavy rainfall [5,23,24,25,26]. Outdoor activities such as freshwater swimming, canoeing, kayaking and triathlons lead to potential exposure to pathogenic *Leptospira* spp. through contaminated water [27,28,29,30,31]. Due to close contact with soil, natural water sources and farm animals, workers on dairy farms and slaughterhouses, harvesters and veterinarians are also at a higher risk of exposure [24,32,33,34]. Pet animals, especially rats, are also a potential source of infection [35].

Accidental hosts, e.g., humans, can develop serious clinical outcomes ranging from unspecific, mild flu-like symptoms to severe clinical onset with fever, jaundice and dysfunction of multiple organs [24,36,37,38,39]. Overall, 5–6.5% of confirmed leptospirosis cases in humans are fatal [4,6]. Due to the unspecific clinical symptoms, leptospirosis is suspected to be highly underdiagnosed in humans [24,40]. The similarity of leptospirosis symptoms to that of malaria, yellow fever and hantavirus disease makes their differentiation difficult and may cause misdiagnosis, especially in tropical regions [4,36].

Because of their wide distribution and potentially close contact to humans, small mammals play an important role in the transmission of *Leptospira* spp. [24,41,42,43,44]. Former studies showed a wide distribution of *Leptospira* spp. in rodents of different genera in Europe. Prevalence ranges from 5.3% in The Netherlands [45], 7.3% in Austria [46], 10.4% in Corsica, France [47], 3.1–12% in Czech Republic [48], 7.9–12% in Spain [49,50] and up to 21.5% in Croatia [51]. In Germany, a mean prevalence of 6% up to 21.3% in rodents of different genera was detected [52,53,54,55,56]. Studies including multiple rodent genera concluded that rodents of the genus *Microtus*, as the main reservoir of *Leptospira*, showed much higher *Leptospira* prevalences (up to over 30%) compared to bank voles (4–11%) [52,57,58]. Further, it is commonly assumed that *Leptospira* species or serovars are adapted to certain host species, e.g., *L. interrogans* serovar Icterohaemorrhagiae is often associated with Norway rats (*Rattus norvegicus*) [59] or *L. kirschneri* with field voles (*Microtus agrestis*) and common voles (*Microtus arvalis*) [54]. In contrast, multiple *Leptospira* genomospecies were detected in the bank vole (*Clethrionomys glareolus*), yellow-necked field mouse (*Apodemus flavicollis*) and wood mouse (*Apodemus sylvaticus*) [58].

The bank vole is widely spread in multiple, but mainly forest habitats in Germany. The population size fluctuates with peaks every two to three years, through bottom-up control of food resources mainly driven by the beech mast of the previous year [60]. The epidemiology of *Leptospira* spp. within bank vole populations is unclear. Our study was conducted to fill this knowledge gap and therefore we aimed to (1) analyze the *Leptospira* spp. prevalence in bank voles at the transect North Rhine-Westphalia and Lower Saxony, Germany; (2) identify *Leptospira* genomospecies and sequence types; and (3) investigate the influence of individual characteristics such as weight and sex as well as season and bank vole abundance on *Leptospira* spp. prevalence in bank voles over three consecutive years.

## 2. Materials and Methods

### 2.1. Sample Collection

**Figure 1:**
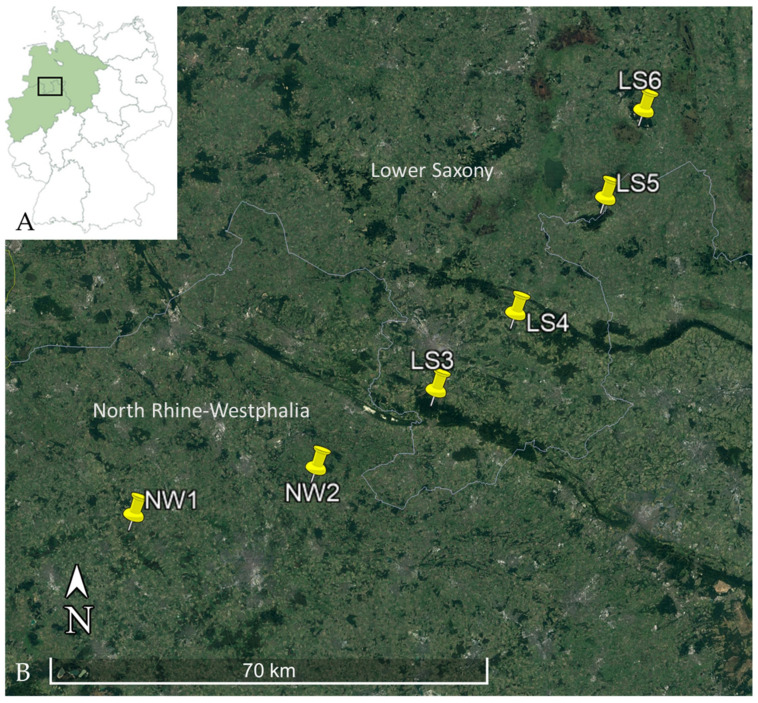
(**A**) Map of Germany with involved federal states highlighted in green and location of the transect marked by a square, (**B**) Trapping locations within the transect, NW = North Rhine-Westphalia, LS = Lower Saxony. Image was created by using Google Earth Pro, Map: Google Earth ©2021 Google, Image Landsat/Copernicus ©2021 GeoBasis-DE/BKG.

Bank voles were trapped during 2018–2020 along a transect for Puumala orthohantavirus (PUUV) monitoring within the RoBoPub consortium covering parts of North Rhine-Westphalia and Lower Saxony [61]. The locations in North Rhine-Westphalia (NW1 and NW2) and Lower Saxony (LS3, LS4, LS5, LS6) were chosen because of high human hantavirus incidences and to find the border of the PUUV distribution in bank voles (Figure 1). Live and snap traps were set in small beech forests in North Rhine-Westphalia; carcasses from snap traps and animals that were found dead in live traps were included in this study (Table A1). Collection of bank voles in Lower Saxony was performed exclusively by snap trapping (Figure 1, Table A1).

At each trapping location, 49 or 100 traps were set for three consecutive nights at multiple sites (2–13 sites per location) (Table A1). Trapping success was documented once or twice a day (Table A1).

During dissection, species, weight, and sex were recorded. The obtained kidney tissue samples were stored at −20 °C until further investigation.

The relative abundance index was calculated by standardizing the number of trapped bank voles into individuals per 100 trap nights for each trapping session.

### 2.2. DNA Extraction

For DNA extraction, one kidney of each animal was used. Depending on the size of the kidney, 10 to 180 mg of tissue were mixed with a fourfold amount of phosphate-buffered saline (PBS, at least 160 µL) and placed in a vial with 0.6 g of 1.4 mm-sized zirconium oxide beads (Bertin Technologies SAS, Montigny-le-Bretonneux, France). Samples were homogenized by using Precellys^®^ 24 lysis & homogenization (Bertin Technologies SAS, Montigny-le-Bretonneux, France) and two rounds of shaking at 5500 rpm with a 10 s break. In total, a 125 µL-aliquot of each sample was used for further extraction.

DNA extraction was performed using the QIAamp DNA Mini Kit (Qiagen, Hilden, Germany) following the manufacturer’s instructions.

The quality of the DNA preparations was controlled by measuring with a spectrophotometer (PEQLAB Biotechnologies GmbH, Erlangen, Germany).

### 2.3. PCR Methods and Multilocus Sequence Typing

Initially, all samples were tested by quantitative PCR (qPCR), targeting a fragment of the *lipl32* gene (242 base pairs, bp) (Table A2) encoding an outer membrane lipoprotein. The PCR followed the protocol by Stoddard et al. [62] and was performed using the Qiagen QuantiTect Multiplex no Rox Kit (Qiagen, Hilden, Germany) and the Agilent Mx3000P qPCR System (Agilent, Santa Clara, CA, USA). The DNA of a laboratory strain of *L. kirschneri* serovar Grippothyphosa was used as a positive control.

Only samples with a sufficient amount of DNA were included in the typing analyses. Based on experiences from prior studies, samples showing a Cycle threshold (Ct) value below 35 in screening qPCR were analyzed by SLST to determine the *Leptospira* species. The DNA concentration of the samples was adjusted to 40–80 ng/µL and the PCR protocol by Victoria et al. [63] detecting the *secY* gene (657 bp) was used (Table A2). We slightly modified the protocol by using the HotStarTaq DNA Mastermix (Qiagen, Hilden, Germany). PCR products were visualized by electrophoresis on 2% agarose gels stained with HDGreen Plus DNA Stain (Intas Science Imaging Instruments GmbH, Göttingen, Germany). The DNA of a laboratory strain of *L. interrogans* serovar Icterohaemorrhagiae was used as a positive control.

The PCR products were purified using the Invisorb Fragment CleanUp Kit (Invitek, Berlin, Germany) or Macherey-Nagel Nucleo Spin Gel and PCR Clean-up (Macherey-Nagel, Düren, Germany) following the manufacturer’s instructions. Sequencing was commercially performed using forward and reverse primers of the performed PCR (Interdisziplinäres Zentrum für Klinische Forschung, Leipzig, Germany and Eurofins Genomics Germany GmbH, Ebersberg, Germany). Bionumerics (Applied Maths NV, Sint-Martens-Latem, Belgium) was used to assemble the resulting sequences.

*Leptospira* species were identified by comparing the resulting sequences with reference sequences of 127 *Leptospira* isolates using the online Basic Local Alignment Search Tool (BLAST) (https://blast.ncbi.nlm.nih.gov/Blast.cgi). The assignment of genomospecies was confirmed if the percent nucleotide sequence identity was 100%.

MLST was performed for *secY*-positive samples with a Ct value less than 28 in the screening qPCR. The MLST scheme by Boonsilp et al. was used, and detected the following genes: *glmU* (expected size of amplicon: 650 bp), *pntA* (621 bp), *sucA* (640 bp), *tpiA* (639 bp), *pfkB* (588 bp), *mreA* (791 bp), *caiB* (650 bp) (Table A2) [2]. PCR was performed in a volume of 25 µL per reaction, containing 0.625 unit GoTaq^®^ G2 Flexi DNA Polymerase, 1 unit Green GoTaq^®^ Flexi Buffer, 1.5 mM MgCl_2_ (Promega High-Performance GoTaq^®^ G2 DNA Polymerase with Mg-Free Buffer System, Promega, Madison, WI, USA), 200µM dNTP (Thermo Fisher Scientific, Waltham, MA, USA), 5pmol of each forward and reverse primer and 2 µL DNA preparation. Amplification was performed according to Boonsilp et al., with slightly modified to 35 cycles of annealing with an annealing temperature of 50 °C. PCR products were visualized by gel electrophoresis on 1.2% agarose gels stained with GelRed^®^ Nucleic Acid Gel Stain (Biotium Inc., Fremont, CA, USA) or HDGreen Plus DNA Stain (Intas Science Imaging Instruments GmbH, Göttingen, Germany). DNA of a laboratory strain of *L. interrogans* serovar icterohaemorrhagiae was used as positive control.

Bionumerics was used to analyze the allelic profile of each gene, using the trimming patterns provided by PubMLST (https://pubmlst.org, accessed on 18 August 2020). The sequence type was identified by the combination of allelic profiles using the PubMLST database (https://pubmlst.org, accessed on 20 August 2020).

### 2.4. Statistical Analyses

Confidence intervals (95% CI) for prevalence of *Leptospira* spp. in bank voles were determined by the Clopper and Pearson method with Graph Pad Software (Graph Pad Software Inc., San Diego, CA, USA).

Independence of compared sample sizes was tested with the two-tailed chi-squared test and a significance level of α = 0.05 and Yates correction for comparison of prevalence of *L. interrogans* versus all positive tested samples in *lipl32* qPCR and *L. interrogans* versus all samples tested.

To analyse *Leptospira* spp. prevalence variations within bank vole hosts, we generated generalised linear mixed models (GLMM) using the *lme4* package [64] within the R-software [65]. The infection status of individuals was used as a binary dependent variable (either *Leptospira* spp. positive/negative) giving the GLMM a binomial error structure. In total three separate GLMMs were generated to estimate how (1) seasonality; (2) individual demographics; and (3) direct and delayed host abundance can influence the probability of individual infection. The following independent variables were incorporated: (1) season (categorical; spring, summer, autumn) and year (categorical; 2018, 2019, 2020) as well as the interaction between season and year to estimate seasonality within each year. As the interaction term consisted of two variables with three levels each, we estimated marginal means using the *emmeans* package and compared within-subject contrasts for each year (post-hoc analysis) [66]; (2) Sex (categorical; male/female), weight (continuous; in gram body weight [g]); and (3) Bank vole abundance of the present season (abundance) and abundance of the previous season (delayed abundance). In addition, study site as well as season nested in year were incorporated as random factors to account for the spatially and temporally replicated study design. All analyses were performed in R base version 4 [65].

## 3. Results

### 3.1. Collection of Rodents

In total, 1817 bank voles were trapped in the transect during the years 2018 (*n* = 263), 2019 (*n* = 1116) and 2020 (*n* = 438) (Table 1). During dissection, all animals were considered healthy and no macroscopic lesions were documented.

There were 972 males (53.5%) and 779 females (42.8%). For 66 voles, sex could not be determined due to poor sample condition; these samples were excluded from further statistical analyses regarding the influence of sex on the individual infection probability.

### 3.2. Leptospira Prevalence, Genomospecies and ST Determination

Overall, 137 samples out of 1817 were *Leptospira* spp. positive in the *lipl32* qPCR (7.5%, 95% CI: 6.4–8.9). Detection of *Leptospira* genomospecies by *secY*-SLST was possible for 96 samples (Table 1) (GenBank accession numbers: MZ678532-MZ678627).

*L. interrogans* (83.3%, 95% CI: 74.4–90.2) was the most frequently detected *Leptospira* genomospecies out of all samples tested for *Leptospira* spp. DNA (χ^2^ = 42.466, df = 1, *p* < 0.0001) and out of all samples tested positive in *lipl32* qPCR (χ^2^ = 63.642, df = 1, *p* < 0.0001). The genomospecies *L. kirschneri* (11.5%, 95%CI: 5.9–19.6) and *L. borgpetersenii* (5.2%, 95% CI: 1.7–11.7) were less often detected.

MLST was possible for 36 samples and *L. interrogans* ST24 (*n* = 29), *L. kirschneri* ST110 (*n* = 4) and *L. borgpetersenii* ST197 (*n* = 3) (Table 1) were identified. Sequence types are related to certain serogroups and serovars as follows: ST24 is related to serogroup Australis serovar Bratislava, Jalna, Lora, Muenchen; ST110 is related to serogroup Grippotyphosa serovar Grippotyphosa, Vanderhoedeni, Valbuzzi; and ST197 is related to serogroup Sejroe [67].

### 3.3. Influence of Seasonality, Body Weight, Sex and Abundance on the Probability of Infection

**Figure 2:**
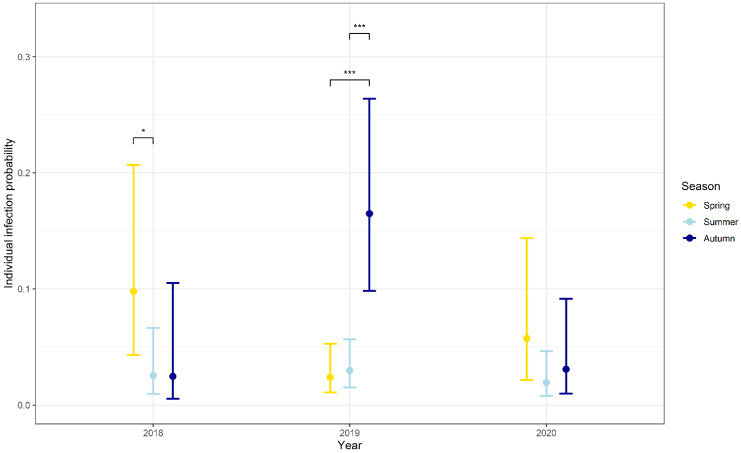
Potential influence of season on the individual infection probability in the years 2018 to 2020. Results are based on *lipl32*-qPCR-positive animals, significant differences between seasons are highlighted by stars: * = significant (*p* < 0.05), *** = highly significant (*p* < 0.001).

Overall, there were clear interannual differences with 2019 showing a significantly higher infection probability compared to 2018 and 2020 (Table 2). Different seasonal patterns were observed between the years (Table 2, Figure 2). In the year 2018, the individual infection probability was significantly higher in spring compared to summer but not autumn. The highest individual infection probability was documented in autumn 2019, which was significantly higher compared to summer 2019 (*p* < 0.001) and spring 2019 (*p* < 0.001) (Table 2). There were no significant differences between the seasons in 2020, though infection probability was highest in spring.

**Figure 3:**
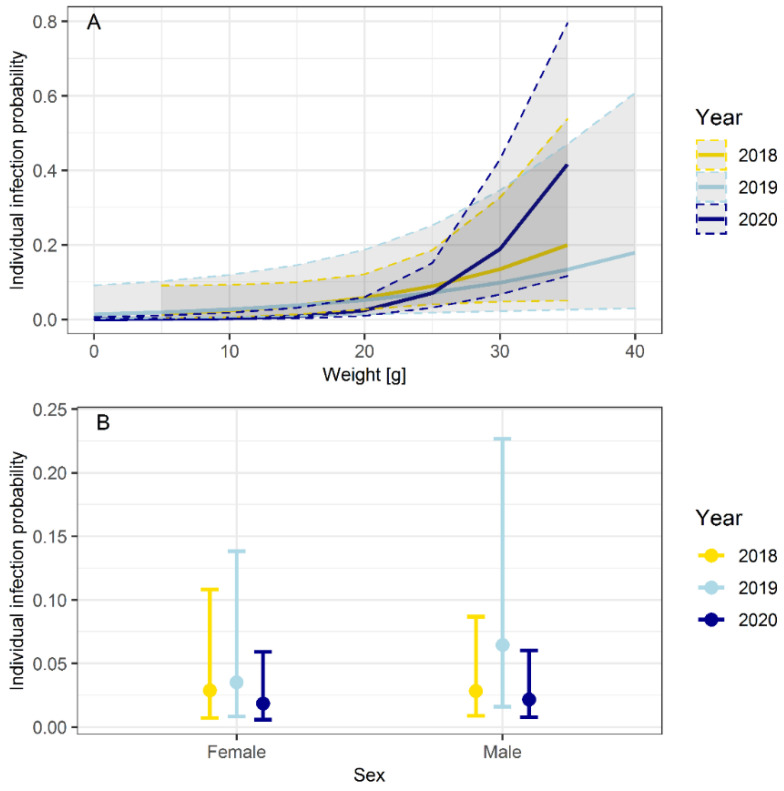
Potential influence of (**A**) body weight and (**B**) sex on individual infection probability. Results are based on *lipl32*-qPCR-positive animals. Only animals with sex determination (*n* = 1751) were analyzed for influence of sex on the individual infection probability.

The predicted probability of infection was positively correlated with body weight in the years 2019 (*p* = 0.045) and 2020 (*p* < 0.001). The correlation was not significant in the year 2018 (*p* = 0.071) (Figure 3A, Table 3).

Sex had no influence on the individual probability of infection in 2018 (*p* = 0.773) and 2020 (*p* = 0.764). In contrast, male bank voles were more often *Leptospira* spp.-positive than female bank voles in the year 2019 (*p* = 0.015) (Figure 3B, Table 3).

**Figure 4:**
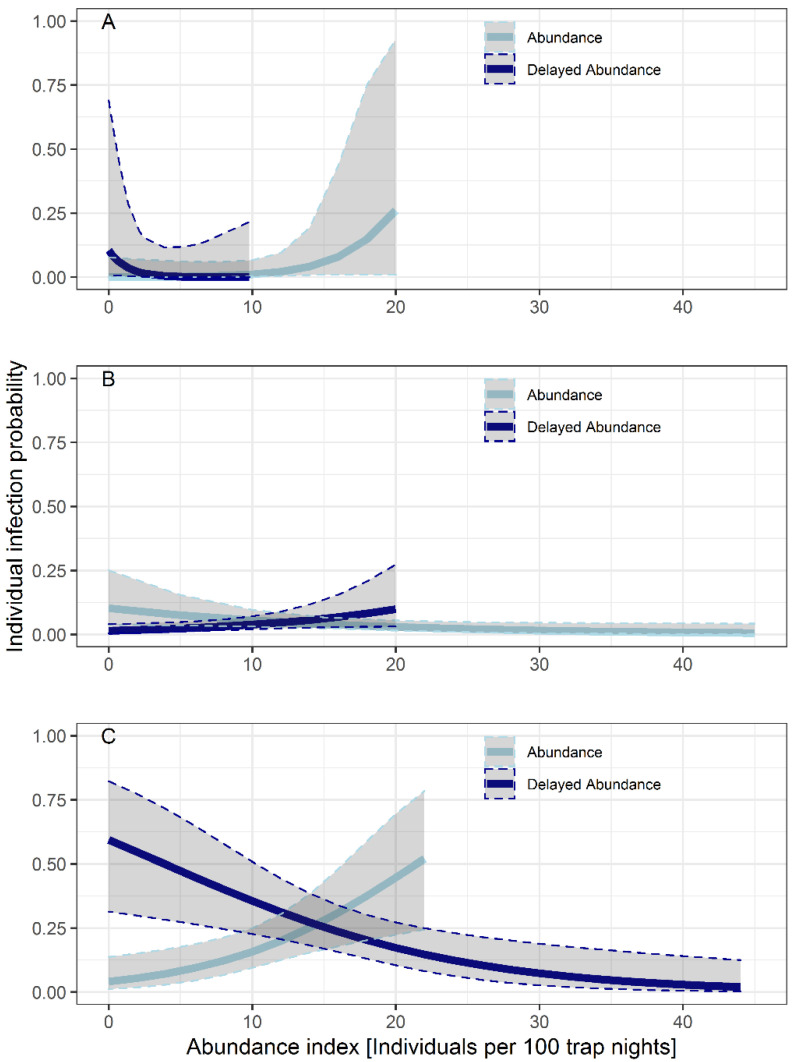
Potential influence of abundance and delayed abundance in (**A**) spring, (**B**) summer and (**C**) autumn in the year 2019 on individual infection probability (Abundance = bank vole abundance of the present season, Delayed Abundance = bank vole abundance of the previous season). Results are based on *lipl32*-qPCR-positive animals. (Years 2018 and 2020 are not shown, because no significant influences were documented).

There was no statistical effect of the abundance on infection probability of bank voles in the years 2018 and 2020. In summer 2019, there was a positive relationship between the individual probability of infection and abundance in the previous season (spring 2019; delayed abundance) (*p* = 0.038) and a negative relationship with increasing abundance of the present season (*p* = 0.042) (abundance) (Figure 4B, Table 4). In the following autumn (2019), the direction of this relationship switched. The infection probability was significantly negatively-related to increased abundances in the previous season (*p* = 0.004) and positively related to abundances in the present season (*p* = 0.005) (Figure 4C, Table 4).

## 4. Discussion

Our study detected a mean *Leptospira* spp. prevalence of 7.5% (95% CI: 6.4–8.9), with a range of 0% (LS6) to 15.7% (NW1) in bank voles from North-West Germany in the years 2018 to 2020 (Table 1). Results of previous studies in Germany showed mean prevalence values of 4.1% (95% CI: 2.8–5.9) up to 11.4% (95% CI: 8.7–14.6%) in bank voles [52,57], which is similar to our results 

However, there are obvious regional differences. Lower values were found in studies investigating the *Leptospira* prevalence in South and East Germany. Here, a prevalence of 0% up to 13% was detected in bank voles, depending on the site and year [53,57]. Another study, which captured different small mammal species at multiple locations throughout Germany in the years 2002 to 2010, detected a mean prevalence of 6% (66/1016) in bank voles [54].

A similar mean prevalence (7.8%) in bank voles was detected across four locations in Germany in the years 2010 to 2014 [58]. One trapping site of the former study in North Rhine-Westphalia is in close geographical proximity to NW1 of our study, and here the authors found a slightly higher prevalence (20.4%, 95% CI: 16.1–25.2) [58] compared to our study (15.7%, 95% CI: 12.9–18.9). A higher mean prevalence in bank voles was also detected in a study in central Germany (11.4%) [52] and the Czech Republic (12%) [48]. The lower mean prevalence in our study might be explained by the extreme drought in the years 2018 to 2020 [68], which gave *Leptospira* a less favorable environment in which to survive [69,70]. On the other hand, it needs to be taken in account that the highest prevalence in our study was documented in 2019, also a year affected by drought (Figure 2), which is most probably due to high bank vole abundance. Further studies need to be done to predict the influence of drought on the *Leptospira* prevalence in bank voles. The absence of *Leptospira* spp. at location LS6 might be due to geographical differences, which inhibited the spread of *Leptospira* spp. in this region. However, the number of collected bank voles (*n* = 60) was rather small.

Data of the genomospecies distribution in bank voles in Europe are rare. Former studies in Germany showed that bank voles can be infected by different *Leptospira* species, with *L. kirschneri* being the most common [52,58]. Our study confirms this observation, but interestingly the most common *Leptospira* genomospecies in bank voles of our study was *L. interrogans*, followed by *L. kirschneri* and *L. borgpetersenii*. These results suggest that bank voles are susceptible to at least three *Leptospira* genomospecies.

In former studies of bank voles, *L. interrogans* was represented by ST24, *L. kirschneri* by ST110 and ST117 and *L. borgpetersenii* by ST197 and ST146 in Germany [53,58]. The findings of our study are in line with these results, except for ST117 and ST146, which were not identified in our study. Certain STs and related serogroups can be associated with a specific host, for example the ST17 serogroup Icterohaemorhagiae with Norway rats [59]. We did not observe a host specificity of serogroups and STs for bank voles in our study, as the STs detected in our study were also found in other small mammal species: ST24 was found in the yellow-necked field mouse, the wood mouse, and the Eurasian beaver (*Castor fiber*); ST110 in the yellow-necked field mouse, common vole, field vole and the common shrew (*Sorex araneus*); and ST197 in the common shrew and crowned shrew (*S. coronatus*) [53,58,71]. Studies from Europe and Asia also show a high ST diversity among different genera of small mammals [72,73,74,75]. Serogroups detected in our study were detected worldwide in different host species, e.g., serogroup Australis in swine, goats, cats and dogs [76,77,78,79]; serogroup Grippotyphosa in cattle, sheep, wild boars, dogs and horses [16,80,81,82,83]; and serogroup Sejroe in cattle [84,85].

Habitat seems to have an important influence on the distribution of different *Leptospira* genomospecies and STs. In previous studies, common voles and field voles, which have their main habitat in grassland, were primarily infected by *L. kirschneri* ST110 [54,58]. In contrast, bank voles, wood mice and yellow-necked field mice, mostly found in forests, were susceptible to *L. kirschneri* ST110, *L. interrogans* ST24, *L. borgpetersenii* ST197, and ST146 [58], which is in line with results of our study.

The STs in our study can be associated with various *Leptospira* serovars. ST24 is associated with serovars Bratislava, Muenchen, Lora and Jalna and ST110 with serovars Grippotyphosa, Vanderhoedenii and Valbuzzi [67]. The ST 197 is related to serogroup Sejroe, but no designated serovar is documented for ST197 [67], as it contains a non-standard length allele (*caiB*51) [2]. Awareness in a public health context should be raised to the pathogenic serogroups Australis, Grippotyphosa and Sejroe and related serovars Bratislava and Grippotyphosa, which can cause severe clinical symptoms in humans and reduced productivity in livestock [8,16]. The finding of serogroups, which are pathogenic for multiple species, underlies the importance of bank voles as reservoirs for *Leptospira* spp. and sources of infection for humans and livestock.

Individual and population-based factors are known to influence *Leptospira* spp. prevalence in rodents. Age is a significant driver of *Leptospira* prevalence in different rodent reservoirs, because the persisting infection with *Leptospira* spp. increases the probability of infection with the increasing age of the host [86,87,88,89]. Our study confirms this observation for bank voles. Taking body weight as a proxy for age, we showed that with increasing body weight, and therefore increasing age, the probability of infection was positively correlated (Figure 3A, Table 3). This effect was more or less consistent of the study period. Although it was not formally significant in 2018 (*p* = 0.071), it was significant in the years 2019 (*p* = 0.045) and 2020 (*p* < 0.001) (Table 3).

The influence of sex on the individual infection probability depended on the collection year. No difference between male and female bank voles was observed in the years 2018 and 2020. In contrast, male bank voles showed a significantly higher prevalence than female bank voles in the year 2019 (*p* = 0.015) (Table 3). This result suggests that sex only had an influence in years with high bank vole abundance. Higher abundance and reproduction causes an increase in male aggressive behavior and contact to multiple sexual partners during the breeding season [90,91], which increases the risk of direct or indirect contact to *Leptospira* spp. However, our results are in line with previous studies, which yielded mixed results as some found no effect of sex [45,47] and others did [92,93].

Overall, effects of body weight (age) were more consistent compared to sex in terms of individual infection probability for *Leptospira* spp. in bank voles. These results are in line with conclusions of former studies [20,58], and has led to the assumption that weight is a good indicator to predict the individual probability of infection with *Leptospira* spp. in bank voles. However, that weight depends on individual developmental circumstances regarding food resources, environmental influences and seasonal fluctuation, and can only be taken as a proxy for age [94,95].

In previous studies, *Leptospira* prevalence increased in common vole and field vole populations over the course of the year with a high prevalence in autumn [52,58]. However, our bank vole study yielded mixed results in terms of seasonality, with annual differences. Seasonal patterns could be detected in the years 2018 and 2020 when the *Leptospira* spp. prevalence was low in summer and high in spring and autumn (Figure 2). An explanation might be the seasonal differences in the composition of the bank vole population. In spring, the population consisted mainly of adult rodents. These overwintering adults, which were persistently infected from the previous season, may have transmitted *Leptospira* spp. to the following spring population and caused high prevalences in spring. During the breeding season in summer, uninfected juveniles entered the population, which lead to lower prevalences in summer. This “juvenile dilution effect” was also described for PUUV infections in bank voles [96]. Additionally, maternal antibodies transmitted from infected females to their offspring might lead to lower prevalences [97]. In autumn, at the end of the breeding season, adults that dominated [98] were more likely to be infected (Figure 3A, Table 3). Furthermore, seasonal weather conditions can have an impact on *Leptospira* prevalence. The survival of *Leptospira* spp. outside the host is dependent on humidity and water availability, temperature and pH [21]. In summer, higher temperatures and dry conditions may have led to decreased indirect transmission and therefore a lower risk of infection compared to spring and autumn.

In contrast to the described seasonal patterns in the years 2018 and 2020, a different course was documented in the year 2019, when prevalence increased throughout the year, with the highest prevalence in autumn (23.0%, 95% CI: 18.3–28.3). The prevalence in autumn 2019 was the highest documented in the three years of our study (Figure 2, Table 2). This might have been due to high bank vole abundance in 2019, which was caused by the beech mast in the year 2018 [99]. This result was also reflected in high PUUV prevalence in bank voles in North-West Germany in the year 2019 [99]. High bank vole abundance should have promoted high *Leptospira* prevalence in autumn 2019. In summer, the prevalence increased in regions which already showed a high abundance in spring. However, even more significant was the influence of abundance in autumn 2019, as the prevalence increased at all locations with high abundance at the present season (*p* = 0.005) (Table 4). As population density rose, direct transmission was promoted, because of more aggressive encounters between conspecifics for food, territory and mates [90,91]. Additionally, more leptospires were potentially shed into the environment via the urine of persistently infected bank voles that frequently mark their home range by shedding urine [91], which can promote indirect transmission through contact with contaminated water and soil.

The influence of abundance to the infection probability differed between season and year. No statistical effect could be documented in the years 2018 and 2020. This result led to the assumption that abundance has an effect on the individual infection probability for *Leptospira* spp. in bank voles, but only during certain seasons and years with high abundance and intense reproduction.

## 5. Conclusions

Our study showed that *Leptospira* spp. are widely spread in bank voles in North-West Germany. The individual infection probability of bank voles was influenced by season, body weight, and year.

This is relevant for public health, as the three detected *Leptospira* species were classified as pathogenic [3], and the detected STs were related to pathogenic serovars. People working in agriculture and forestry are at a particularly higher risk because of their close contact to small mammals, natural water sources and soil.

The human incidence in Germany was low (0.06–0.2 per 100,000 inhabitants) in the years 2010 to 2020, but leptospirosis should be on the differential diagnosis list of every clinician and veterinarian. Incidence may rise due to increasing popularity of outdoor activities, which lead to a higher risk of exposure in the human population. Furthermore, climate change might be an additional influencing factor as it will lead to more extreme weather events in moderate climate zones, like drought, storm, heavy rainfalls and flooding [100], as recently seen in Germany in the summer of 2021.

## Figures and Tables

**Table 1:** *Leptospira* spp. prevalence and genomospecies distribution in bank voles captured in the years 2018 to 2020.

Federal State	Total Number of Bank Voles	Trapping Location (see Figure 1)	Number of *Leptospira* DNA Positive/Total Number of Bank Voles Tested (*lipl32*-qPCR) (Percentage, 95% CI)	Number of *secY*-SLST/MLST Positive Bank Voles *		
				*L. interrogans/* ST24	*L. kirschneri/* ST110	*L. borgpetersenii/* ST197
North Rhine-Westphalia	782	NW1	95/605 (15.7%, 12.9—18.9)	55/15	8/3	2/1
		NW2	12/177 (6.8%, 3.6—11.5)	7/5	1/0	N/A
Lower Saxony	1035	LS3	1/160 (0.6, 0—3.4)	N/A	N/A	N/A
		LS4	26/770 (4.3%, 2.2—4.9)	17/9	2/1	3/2
		LS5	3/45 (6.7%, 1.4—18.3)	1	N/A	N/A
		LS6	0/60 (0%, 0—6%)	N/A	N/A	N/A
total	1817		137/1817 * (7.5%, 95% CI: 6.4—8.9)	80/29	11/4	5/3

NW = North Rhine-Westphalia, LS = Lower Saxony, CI = confidence interval, SLST = single locus sequence typing, MLST = multi locus sequence typing, ST = sequence type, N/A = not available, * Determination of genomospecies and ST was not possible for all samples tested positive in *lipl32*-qPCR.

**Table 2:** Results of generalized linear mixed modelling with binomial error distribution showing the influence of season, year and their interaction on the individual infection probability.

Factor	Estimate	Std. Error	z-Value	*p*-Value
Intercept	**−3.676**	**0.783**	**−4.696**	**<0.001**
Season [spring]	1.454	0.823	1.767	0.077
Season [summer]	0.032	0.857	0.037	0.971
Year [2019]	**2.054**	**0.757**	**2.712**	**0.007**
Year [2020]	0.225	0.909	0.248	0.804
Year:season	Estimate	Std. Error	z-ratio	p-value
Year 2018				
Autumn v. spring	−1.454	0.823	−1.767	0.181
Autumn v. summer	−0.032	0.857	−0.037	0.999
Spring v. summer	**1.423**	**0.567**	**2.511**	**0.032**
Year 2019				
Autumn v. spring	**2.087**	**0.364**	**5.732**	**<0.001**
Autumn v. summer	**1.868**	**0.285**	**6.554**	**<0.001**
Spring v. summer	−0.219	0.400	−0.546	0.848
Year 2020				
Autumn v. spring	−0.649	0.691	−0.939	0.616
Autumn v. summer	0.473	0.643	0.735	0.743
Spring v. summer	1.122	0.582	1.928	0.131
Random effects	Variance	Std.Dev.		
Site	1.460	1.208		
Year	0.000	0.000		
Season:year	0.000	0.000		

For categorical factors the reference categories are: Season—autumn, Year—2018. For the interaction term Year:season the results of a post-hoc (Tukey) analysis on the estimated marginal means are presented to highlight seasonal differences within each year. Bold values represent significant factors. Std. Error = Standard Error; Std.Dev. = Standard Deviation, v. = versus, z-value/ratio = Wald statistics.

**Table 3:** Results of generalized linear mixed modelling with binomial error distribution showing the impact of demographic factors on individual infection probability for each year.

Factor	Estimate	Std. Error	z-Value	*p*-Value
Year 2018				
Intercept	**−4.746**	**1.277**	**−3.717**	**<0.001**
Sex [m]	0.150	0.518	0.289	0.773
Weight	0.093	0.052	1.808	0.071
Random effects	Variance	Std.Dev.		
Site	0.709	0.842		
Season	0.137	0.370		
Year 2019				
Intercept	**−4.639**	**1.039**	**−4.466**	**<0.001**
Sex [m]	**0.637**	**0.262**	**2.433**	**0.015**
Weight	**0.069**	**0.035**	**2.001**	**0.045**
Random effects	Variance	Std.Dev.		
Site	1.614	1.271		
Season	1.323	1.150		
Year 2020				
Intercept	**−8.284**	**1.680**	**−4.931**	**<0.001**
Sex [m]	0.161	0.535	0.301	0.764
Weight	**0.224**	**0.065**	**3.441**	**<0.001**
Random effects	Variance	Std.Dev.		
Site	1.525	1.235		
Season	0.000	0.000		

For categorical factors the reference categories are: Sex—female. Bold values represent significant factors. Std. Error = Standard Error; Std.Dev = Standard Deviation, z value/ratio = Wald statistics.

**Table 4:** Results of generalized linear mixed modelling with binomial error distribution showing the impact of abundance and delayed abundance on individual infection probability for the year 2019.

Factor	Estimate	Std. Error	z-Value	*p*-Value
Spring 2019				
Intercept	**−5.519**	**1.714**	**−3.220**	**0.001**
Delayed abundance	−0.850	0.553	−1.538	0.124
Abundance	0.352	0.219	1.605	0.109
Random effects	Variance	Std.Dev.		
Site	2.802	1.674		
Summer 2019				
Intercept	**−2.845**	**0.602**	**−4.727**	**0.000**
Delayed abundance	**0.102**	**0.049**	**2.078**	**0.038**
Abundance	**−0.068**	**0.033**	**−2.030**	**0.042**
Random effects	Variance	Std.Dev.		
Site	0.398	0.631		
Autumn 2019				
Intercept	**−1.284**	**0.576**	**−2.230**	**0.026**
Delayed abundance	**−0.097**	**0.034**	**−2.865**	**0.004**
Abundance	**0.147**	**0.052**	**2.802**	**0.005**
Random effects	Variance	Std.Dev.		
Site	0.611	0.782		

Abundance = Bank vole abundance of the present season, Delayed abundance = abundance of the previous season. Bold values represent significant factors. Std. Error = Standard Error; Std.Dev = Standard Deviation, z value/ratio = Wald statistics.

## Data Availability

The data presented in this study are available in the article.

## Appendix A

**Table A1:** Details of rodent trapping in transect North-Rhine Westphalia and Lower Saxony, Germany in the years 2018 to 2020.

Federal State	North Rhine-Westphalia		Lower Saxony
**Trapping location** **(Number of trapping sites per** **location)**	NW1 (13 trapping sites)		LS3 (3 trapping sites)
	NW2 (3 trapping sites)		LS4 (6 trapping sites)
			LS5 (2 trapping sites)
			LS6 (2 trapping sites)
**Trapping frequency**	Three times yearly in spring (March to Mai), summer (July, August) and autumn (October to November)		Three times yearly in spring (April), summer (July) and autumn (October)
**Type of trap**	Ugglan multiple capture live traps (Grahnab^®^, Gnosjö, Sweden)	Metal snap traps (Deufa, Neuburg, Germany)	Metal snap traps (Deufa, Neuburg, Germany)
**Traps per night and trapping site/Number of nights/Control** **frequency**	49 traps/ 3 nights/ Checked twice a day	49 traps/ 3 nights/ Checked once a day	100 traps/ 3 nights/ Checked once a day
**Bait**	apple, rodent pellets, rolled oats, peanut curls	peanut butter with rolled oats	peanut butter with rolled oats
**Additional information**	Rodents captured alive were sampled and released, only rodents found dead were used in this study		
**Publication**	Trapping according to [101]	Trapping according to [99]	Trapping according to [99]

**Table A2:** Gene loci, primer sequences and probe used for qPCR [62], SLST [63] and MLST [2].

Locus	Primer/BREAKProbe	Sequence (5′ to 3′)
*lipl32*	lipl32-F	AAG CAT TAC CGC TTG TGG TG
	lipl32-R	GAA CTC CCA TTT CAG CGA TT
	probe	6FAM-AA AGC CAG GAC AAG CGC CG BHQ1
*secY*	secY-F	GAA TTT CTC TTT TGA TCT TCG
	secY-R	GAG TTA GAG CTC AAA TCT AAG
*glmU*	glmU-F	AGG ATA AGG TCG CTG TGG TA
	glmU-R	AGT TTT TTT CCG GAG TTT CT
*pntA*	pntA-F	TAG GAA ARA TGA AAC CRG GAA C
	pntA-R	AAG AAG CAA GAT CCA CAA YTA C
*sucA*	sucA-F	TCA TTC CAC TTY TAG ATA CGA T
	sucA-R	TCT TTT TTG AAT TTT TGA CG
*tpiA*	tpiA-F	TTG CAG GAA ACT GGA AAA TGA AT
	tpiA-R	GTT TTA CRG AAC CHC CGT AGA GAA T
*pfkB*	pfkB-F	CGG AGA GTT TTA TAA RAA GGA CAT
	pfkB-R	AGA ACA CCC GCC GCA AAA CAA T
*mreA*	mreA-F	GGC TCG CTC TYG ACG GAA A
	mreA-R	TCC RTA ACT CAT AAA MGA CAA AGG
*caiB*	caiB-F	CAA CTT GCG GAY ATA GGA GGA G
	caiB-R	ATT ATG TTC CCC GTG AYT CG

F = forward primer, R = reverse primer.
